# Developing and Testing Molecular Markers in *Cannabis sativa* (Hemp) for Their Use in Variety and Dioecy Assessments

**DOI:** 10.3390/plants10102174

**Published:** 2021-10-14

**Authors:** Marcello Borin, Fabio Palumbo, Alessandro Vannozzi, Francesco Scariolo, Gio Batta Sacilotto, Marco Gazzola, Gianni Barcaccia

**Affiliations:** 1 Department of Biotechnology, University of Verona, Strada Le Grazie 15, 37034 Verona, Italy; marcello.borin@univr.it; 2 Gruppo Padana Ortofloricoltura S.S., Via Olimpia 41, 31038 Treviso, Italy; giobatta@gruppopadana.com (G.B.S.); marco.gazzola@gruppopadana.com (M.G.); 3 Department of Agronomy, Food, Natural Resources, Animals and Environment (DAFNAE), University of Padova, Viale dell’Università 16, 35020 Padova, Italy; fabio.palumbo@unipd.it (F.P.); alessandro.vannozzi@unipd.it (A.V.); francesco.scariolo@phd.unipd.it (F.S.)

**Keywords:** SSR, microsatellites, genotyping, geographical origin, dioecious, monoecious

## Abstract

*Cannabis sativa* (2*n* = 2*x* = 20) is a popular species belonging to the Cannabaceae family. Despite its use for medical, recreational, and industrial purposes as well as its long history, the genetic research on this species is in its infancy due to the legal implications and the prohibition campaigns. The recent legalization of *Cannabis* in many countries along with the use of genomics boosted the approaches aimed at marker-assisted selection, germplasm management, genetic discrimination, and authentication of cultivars. Nonetheless, the exploitation of molecular markers for the development of commercial varieties through marker-assisted breeding schemes is still rare. The present study aimed to develop an informative panel of simple sequence repeat markers to be used for the genotyping of high breeding value *C. sativa* lines. Starting from 41 nuclear SSR designated by in silico analyses, we selected 20 highly polymorphic and discriminant loci that were tested in 104 individuals belonging to 11 distinct hemp varieties. The selected markers were successful in accessing homozygosity, genetic uniformity, and genetic variation within and among varieties. Population structure analysis identified eight genetic groups, clustering individuals based on sexual behaviors (dioecious and monoecious) and geographical origins. Overall, this study provides important tools for the genetic characterization, authentication, conservation of biodiversity, genetic improvement and traceability of this increasingly important plant species.

## 1. Introduction

*Cannabis sativa* L. is a multiuse plant species cultivated for its fiber and seeds (var. *sativa*; hemp) or for its high content in cannabinoids (var. *sativa* and var. *indica*) [1]. Among the latter, the principal psychoactive constituent of *Cannabis* is tetrahydrocannabinol (THC) with varieties containing up to 30% THC by dry weight [2]. The genus *Cannabis* belongs to the family Cannabaceae (order Rosales), and its nomenclature has been disputed since the Linnaeus classification in 1753. At that time, it was not clear whether the genus was mono- or polytypic [3,4,5]. Subsequently, Small and Cronquist [4] proposed a unique species system, establishing the existence of two subspecies of *C. sativa*, namely, subsp. sativa and indica, the validity of which is still universally accepted. Recently, some authors have proposed a classification of *C. sativa* varieties based on their cannabinoid and terpenoid profiles [6,7,8]. In contrast to this classification, a method based on DNA markers may be useful to develop a fast and low-cost technique enabling the solution to this taxonomic debate. These markers may also be employed in the identification and characterization of uncertified *Cannabis* strains, including those originating from the black market [9].

From a reproductive point of view, *Cannabis* is prevalent as a dioicous plant although monoecism and even hermaphroditism are possible with plants showing male and female reproductive organs within the same plant and flower, respectively [4,10,11]. All these biological variants are more frequent in industrial hemp varieties [4]. According to Charlesworth et al. (2005), dioecism evolved from a common monoecious ancestor shared by both *Cannabis* and *Humulus* genera [2].

The *C. sativa* genome is composed of nine pairs of autosomes and a pair of sex chromosomes (X and Y). Heterogametic (XY) individuals are males, while females and monoicous individuals are homogametic (XX) [12]. The mechanisms involved in sex determination are still poorly understood because beyond genetic control, they are also influenced by environmental conditions, such as stress factors and chemical treatments [13].

Traditionally, both hemp and drug *C. sativa* varieties were bred through mass selection, optimizing basic quality traits, such as the content of fiber, oil, and cannabinoids [14]. New *Cannabis* cultivars were also constituted by crossing individuals selected from different landraces and cultivars to increase hybrid vigor and, therefore, productive performances. The “Skunk No. 1” hybrid, produced in the early 1970s, represents a successful variety obtained by this conventional breeding approach.

Recently, the advent of genomics applied to crop plant species has revolutionized conventional breeding methods. The use of marker-assisted selection (MAS) allows specific traits of agronomic interest to be controlled, and marker-assisted breeding (MAB) exploits the presence of molecular markers dispersed among the genome for the genetic characterization of samples and the breeding of new varieties [15,16,17]. Molecular markers, as opposed to morphological, biochemical, and cytological markers, have many advantages, including high abundance and wide distribution within the genome as well as a high level of polymorphism and independence from environmental factors [18]. In recent times, molecular markers in *Cannabis* have been mostly employed to investigate genetic diversity, geographical origin, relationships between cultivated germplasm, varietal characterization and sex determination as well as to generate genetic linkage maps [19]. Among molecular markers, SSRs possess numerous desirable features, such as a high level of reproducibility and codominant heredity. Moreover, in contrast to other markers such as SNPs, microsatellites do not require high-throughput technologies and computational resources for their development and analysis [20,21,22].

Microsatellites have been frequently used in *Cannabis* cultivars and genotypes to evaluate genetic diversity. While early studies have identified and characterized a small number of polymorphic SSR sites specific for *Cannabis sativa*, [23,24,25], these markers have been recently used to discriminate between drug- and fiber-type (hemp) varieties [1,13,23,26,27,28,29,30,31], demonstrating their effectiveness as an alternative method to biochemical analyses [2].

Regarding geographical origin, a several studies have reported that the highest genetic diversity is found within hemp varieties compared to drug varieties, and it is possible to differentiate distinct gene pools in Europe and Central Asia [27,32,33].

A previous study has developed a panel of SSR markers, organized in multiplex assays, to predict in silico from polymorphic and widely distributed microsatellite regions of *Cannabis* spp. [9]. However, this SSR panel has never been tested. Starting from the validation of the 41 SSR loci previously identified, the present study evaluated the capability of these markers to discriminate *Cannabis sativa* varieties of high breeding value based on their geographical origin and, possibly, on their sexual attitudes (dioecious and monoecious).

Several experiments have focused on developing a reliable molecular marker for sex determination in *Cannabis* plants. Separating male and female plants at early developmental stages is useful due to the influence of gender on agriculturally significant traits [34]. Sequence-characterized amplified region (SCAR) markers—such as SCAR119—have been developed to identify male plants, which are presented with a single band of 119 bp [35]. However, from the point of view of genetics and breeding, the real challenge is identifying markers linked to the monoecious trait, particularly in view of the current widespread use of monoecious hemp and its better compatibility with modern agriculture strategies. The karyological identity of monoecious plants to female ones [12,36] makes the identification of tightly linked markers difficult [37].

Linkage disequilibrium (nonrandom association of alleles at different loci) is a sensitive indicator of the population genetic forces that structure a genome [38]. In the present study, linkage disequilibrium was used to examine correlations between sex and the developed SSR markers and among the markers themselves.

## 2. Results and Discussion

### 2.1. Overall Genetic Diversity

Based on a previous study on the *C. sativa* (cs10) genome (BioProject ID: PRJNA560384), a total of 126,593 perfect and 12,017 compound SSR regions were identified with a density equal to 148 SSRs/Mbp and 0.34% of the total length of the genome [9]. In total, 41 primer pairs amplifying an equivalent number of SSR loci, with an average of 4 markers per chromosome, were designed in silico and tested on a subset of DNA samples obtained considering at least one sample from each variety. Of these loci, 4 did not produce any amplicon, 6 produced nonspecific products, and 11 were discarded for the high number of missing data among samples. The remaining 20 loci were used to screen on the entire collection, comprised of 104 individuals belonging to 11 different hemp varieties of European origin, both monoecious and dioecious. The SSR profiles of each sample is provided as supplementary material (Table S1).

Table 1 summarizes the number of detected alleles per locus (N), the frequency of the most abundant allele (p_i_), the observed heterozygosity (H_o_), expected heterozygosity (He), average heterozygosity (H_a_) and the polymorphism information content (PIC) of the 20 selected loci.

In total, 301 alleles were detected among 11 varieties with several observed alleles per locus ranging between 3 (SSR_6-4) and 28 (SSR_X-1). According to [39], 16 of 20 SSR loci were highly informative (PIC > 0.5), and 4 SSR loci were reasonably informative (0.5 > PIC > 0.25). It is likely that the high number of alleles per locus (N_a_) and, therefore, the resulting PIC values reflect the extension of the geographical area of varietal origin. A similar average PIC value (0.71) was obtained by [40] by analyzing samples collected from different European regions with 16 SSR loci. In contrast, limiting analyses to samples derived exclusively from Turkey with 22 markers [41] obtains a much lower average PIC (0.28). The frequency of the most common marker allele (p_i_) was low when the observed number of marker alleles was high and vice versa. For instance, SSR_6-4 and SSR_X-1, had Na values equal to 3 and 28, showed pi values of 0.67 and 0.13, respectively.

Although extremely variable (loci ranging from 0.07-SSR_2-1 to 0.84-SSR_8-2), the observed heterozygosity (H_o_), was always lower than the expected heterozygosity (H_e_), highlighting an excess of homozygosity equally distributed across the genome. One possible explanation for this discrepancy is the excess of false homozygotes caused by null alleles. Nuclear SSRs are in fact characterized by high frequencies of null alleles, i.e., alleles that are not amplified due to the failure of primer annealing in the flanking regions of a target SSR locus [42]. Consequently, null alleles are not detected in the expected heterozygous form and the genotypes are incorrectly scored as homozygotes. The occurrence of these alleles can be inferred (and their proportions quantified) in Hardy–Weinberg (HW) equilibrium natural populations or in progenies where parent genotypes are known [42]. In our study, the varieties used do not meet the criteria required for null alleles estimate. Beyond the low size of each variety group (3 < *n* < 13) and the lack of both parents and offspring genotyping data, the main limitation is the lack of HW equilibrium due to the strong selection pressure imposed by breeders. Moreover, the accessions analyzed for each variety are unrelated and were chosen as representative of the variety itself. A second plausible explanation is that the excess of homozygosity is the result of a strong selection pressure and inbreeding, which has characterized hemp cultivation over the years. Industrial hemp is in fact grown on a wide scale leading to a stabilization of agronomically important traits in seed stocks and, likely, to a reduced heterozygosity. On the contrary, drug-type varieties, being the results of hybridization events and being mainly propagated via cloning, are usually characterized by higher levels of heterozygosity [13,43].

In support of this, the inbreeding coefficient (F_is_) result was always positive and, on average, equal to 0.41 (Table 1). Similarly, F_it_ (heterozygosity between subpopulations) and F_st_ (fixation index) indices were highly variable with values ranging from 0.10 to 0.89 and from 0.06 to 0.37, respectively. N_m_ (gene flow measure) ranged from 0.23 to 2.78 with an average value of 1.02 ± 0.13, showing the presence of gene flow (N_m_ > 1) for 9 loci out of 20. Table 2 summarizes the statistics related to the 104 accessions analyzed in the present study, and they are grouped into the 11 varieties of *C. sativa* sampled.

The number of polymorphic loci was high among all varieties analyzed (at least 90% polymorphic loci), except for the FRA3 variety. Nevertheless, it should be emphasized that this result may be affected by the reduced number of individuals considered in this variety compared to the others (only 3 in contrast to the 8–13 of the other varieties). A similar result was observed for the Shannon index (I) of phenotypic diversity and simple matching-based genetic similarity, which showed high values in all varieties (1 to 1.5 and 63% to 69%, respectively), except for the FRA3 variety.

On the other end, the mean genetic similarity (MGS) among all varieties was more uniform with values ranging between 86% and 91%. The mean number of observed and effective alleles per locus ranged from 2.10 to 6.15 and from 1.89 to 4.43, respectively. Despite the different geographical origins and the different sexual behaviors, the observed heterozygosity was comparable among all varieties, and on average, it was equal to 0.58 ± 0.09, consistent with the allogamous reproductive system. When compared to the expected heterozygosity, all varieties showed an excess of homozygosity as confirmed by Wright’s statistics. Both F_is_ and F_it_ were higher than 0 in all varieties, suggesting repeated crosses of genetically related individuals and thus strong selective pressure operated by breeders. Estimates of the fixation index (F_st_) changed considerably (from 0.54 to 0.73) among the 11 varieties, indicating an unbalancing contribution of the investigated populations to the total genetic variation. Accordingly, our estimates of inbreeding coefficients suggested that these varieties are characterized by a relatively high degree of genetic differentiation with approximately 64% (average F_st_ = 0.64) of the genetic variation found among varieties and 36% of the total genetic variation expressed within varieties. As expected, the gene flow (N_m_) estimate derived from F_st_ was low (ranging from 0.09 to 0.21), demonstrating the absence of gene flow among the varieties.

Private alleles, beyond estimating genetic diversity, are also useful for varietal registration and traceability. The 301 alleles were screened for private markers by considering the core collections from different perspectives. The first classification was made between dioecious and monoecious samples, and the second classification considered the 11 varieties as distinct groups. In addition, the third classification was based on the geographical origin (Italy, France, Netherlands, Poland, Hungary and Finland). In the first case, 22 alleles were found to be private for dioecious samples and 13 for monoecious samples. By considering the 11 groups separately, the highest number of private alleles was found in the ITA3 variety (23 out of 123). Finally, the third division of the samples based on their geographical origin found the highest number of private alleles in the Italian, Finnish and Hungarian samples with 13 out of 175, 12 out of 124, and 10 out of 153, respectively. However, it should be noted that the frequency of each private allele was low with few highly frequent alleles. For example, in the case of the ITA3 variety, the private allele frequency ranged from 5% (loci 8–4) to 41.7% (loci 5–2) with 16 out of 23 private alleles having a frequency ≤10%. Although this finding would discourage the use of single SSR loci for traceability or registration purposes, the combination of multiple private alleles at different loci may still represent an excellent tool for varietal identification [44]. The fact that a high number of private alleles was assigned to Italian and Hungarian clusters and varieties suggests a higher degree of isolation and local adaption (or selection) to environmental conditions for the southern samples, and at the same time, it may indicate that this area is the European domestication center of *Cannabis*. In fact, it is conceivable that the longer a species is present in each geographical area, the higher the number of allelic variants (albeit rare) that have accumulated.

### 2.2. Population Structure of Cannabis Germplasms and Cluster Analysis

**Figure 1:**
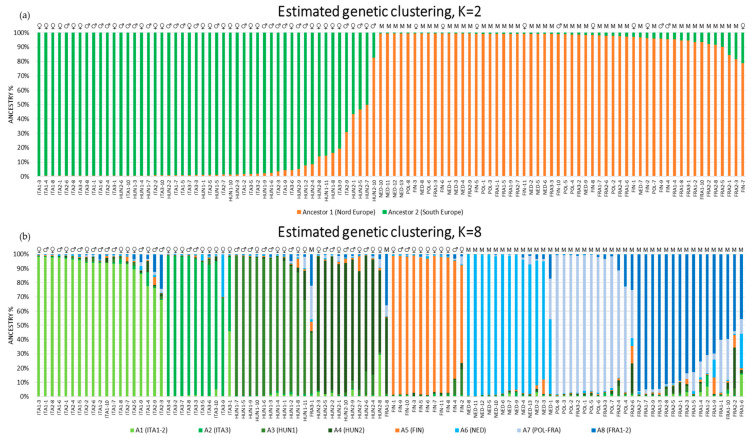
Population structure with K = 2 (**a**) and K = 8 (**b**) of the 104 *Cannabis* accessions based on the 20 selected SSR marker loci. Symbols: ♀: dioecious female sample, ♂: dioecious male sample; M: monoecious sample.

The genetic structure of the germplasm collection was investigated to infer population relationships among individuals (Figure 1).

Following the procedure of [45], a maximum ΔK value was found at K = 2 and K = 8 (ΔK = 181 and ΔK = 23, respectively, Figure S1). For K = 2 (Figure 1, panel a), samples were clearly divided based on geographical origin into two groups as follows: one consisting of varieties from southern Europe (Italian and Hungarian Origin) and the other from northern Europe (France, Poland, Netherlands, and Finland). The clustering of genotypes revealed that 92 of 104 samples showed strong ancestry association (>90%), and on average, the membership of the southern and northern samples to their ancestral group was 92.3% and 96.9%, respectively. In the southern samples, 12 showed admixed ancestry, and in particular, HUN2-10 was the only sample with a higher percentage membership in the opposite group (northern group). Given the geographical distances between these two groups, the most likely hypothesis is that the admixed samples are the intended result of hybridization events among southern and northern genotypes performed in the past by impassionate amateurs or expert geneticists to introduce genetic variability and constitute new lines [9]. It should also be emphasized that the subdivision into two large groups is not only geographic but also based on the reproductive system. In fact, the southern group contained only dioecious plants (i.e., both male and female plants), while the northern group contained only monoecious plants with few exceptions (FIN group). 

**Figure 2:**
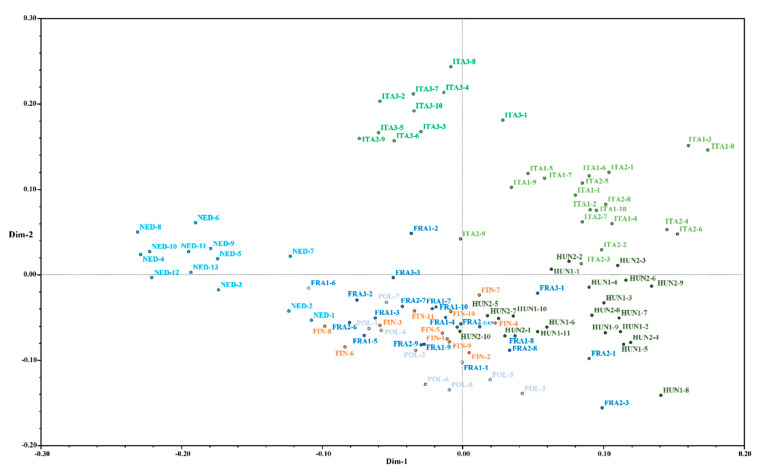
Principal component analysis (PCoA) displaying centroids of individual plants across all *Cannabis* varieties plotted according to the first two dimensions based on the 20 selected SSR marker loci.

Historical information on the introduction of *Cannabis* in Europe is vague. If the center of origin is in Central or East Asia [46], the first data certifying its presence in Europe date back to approximately 10,000 years ago. In particular, paleobotanical evidence shows the presence of wild *Cannabis*/*Humulus*-type pollen in the southeastern part of Europe and, in particular, in Hungary, Romania, and Bulgaria approximately 10200–8500 years BP [46] as well as in Italy (Lake Albano, Rome) 11000 BCE. Although the most accredited hypothesis [47] suggests that dioecious plants (such as those belonging to the southern group in this study) descended from monoecious plants (such as those belonging to the northern group). However, our molecular data along with the paleobotanical evidence mentioned above discourage the hypothesis that the Italian-Hungarian group evolved from the Northern Europe group. It is more likely that the introduction of *Cannabis* in Northern Europe and the Mediterranean basin occurred independently at different times. The clear division between the two European clusters was also clearly visible with PCoA analysis (Figure 2), in which the southern samples were positioned in the top-right part and the northern samples were positioned in the bottom-left part.

When analyzing the germplasm collection with an additional level of population structure (K = 8), sample clustering by geographical area becomes even more detailed. Plant materials from ITA1 and ITA2 were clearly assigned to a single, distinct cluster by STRUCTURE software (Figure 1, panel b) with an average membership value of 91.3%. PCoA (Figure 2) analysis further confirmed this finding with these two varieties visibly separated from the others and occupying the top-right quadrant. Instead, the third group of Italian varieties (ITA3) formed a separate cluster in the structure analysis. This sample group showed the lowest levels of similarity with all the varieties analyzed (Table S2) and clustered separately both in the PCoA (Figure 2) and in the resulting dendrogram (Figure S2). Additionally, the average similarity values with ITA1 (85.4%) and ITA2 (85.1%) were lower than that calculated between ITA1 and ITA2 (88.4%). Since the introduction of *Cannabis* in Italy, it could be hypothesized that the ITA3 group has remained moderately isolated both from the ITA1/ITA2 group and from all other European varieties. This hypothesis was further supported by the number of private alleles found in ITA3, which was the highest among the 11 varieties.

In particular, the HUN1 and HUN2 dioecious samples were assigned to two different clusters by STRUCTURE software. Members of the HUN1 variety showed, on average, higher membership percentages to the cluster (91.9%) compared to the HUN2 ones (85.3%), which was clearly visible in the PCoA with the HUN1 samples tightly clustered in the bottom-right quadrant and the HUN2 samples dispersed around the previous ones. Similarly, samples from Finland (FIN), Poland (POL), and Netherlands (NED) were assigned to three different clusters with an average membership equal to 92.6%, 93.2%, and 91.2%, respectively. The results observed for the three French varieties (FRA1, FRA2, and FRA3) were unexpected. FRA1 and FRA2 formed a cluster apart with an important share of admixed samples and a consistent representation of the Polish ancestor. The only exception was represented by FRA1-8 that resulted admixed also with the Hungarian cluster HUN2 and towards which it showed a mean GS of 88% (Table S2). Similarly, within the FRA3 variety, FRA3-1 was admixed, with a consistent representation of the Hungarian cluster HUN1 (the mean GS between FRA3-1 and HUN1 group was 89%, Table S2); FRA3-2 and FRA3-3 samples clustered instead with high ancestry membership (96.4% and 93.1%, respectively) with the Polish cluster. The close phylogenetic relationship existing between French and Polish samples was also evident from the PCoA and from the similarity matrix. The three French groups showed an average degree of similarity with the POL group oscillating between 86.6% (FRA2) and 88.3% (FRA3). In support of this, a recent study conducted on the occurrence of the CBDA-synthase gene (CBDAS), THCA-synthase gene (THCAS) and two CBDAS pseudogenes across 110 *Cannabis* accessions has demonstrated the tight relationship existing among three French and two Polish genotypes, suggest a recent common ancestor [48]. On the contrary, those samples proving an admixed ancestry between French and Hungarian clusters (i.e., FRA1-8 and FRA3-1) could be the results of hybridization events that have never been described in previous studies.

Overall, this is the first study in which a robust and novel panel of SSRs has been successfully used to investigate the geographical origin of samples derived from different European countries. Previous studies have mainly concerned Asian accessions [49], while the only other study focusing on accessions collected from the same countries considered here (i.e., Italy, Hungary, France, and Finland) failed to reconstruct the geographical origin of the samples [50].

### 2.3. Sex Determination and Linkage Disequilibrium

Separating male and female plants at the seedling stage is useful in breeding programs because plant gender influences the economic value and the selection schemes, which is particularly true in *Cannabis*. Thus, the entire germplasm was screened using UBC354151 RAPD-deriving SCAR119, originally developed by Törjék [35] and named male-associated DNA from *Cannabis* (MADC4). In the last few years, several sex predictive markers have been developed and tested [51], but MADC4 is still considered the most reliable marker [34]. Similarly, our study confirmed that SCAR was 100% effective in predicting the sex of the samples as a 119 bp amplicon was detected in all the male individuals but not in female and monoecious plants. Moreover, to investigate any possible pattern of disequilibrium, we analyzed the association existing between the MADC4 marker locus and each of the 20 selected SSR loci in all possible pairwise combinations. Significant (*P*, 0.01) linkage disequilibria were found between the SCAR119 sex determination locus and the 2.1 and 5.5 SSR loci (Table 3), attributed to chromosomes 2 and 5, respectively.

In the scientific literature, there is no information regarding the localization of the SCAR119 marker as its identification precedes the sequencing of the *Cannabis* genome. However, by aligning the SCAR119 sequence (AB021659.1) on the *Cannabis* reference genome (GCA_900626175.2, cs10), we located MADC4 on chromosome 2 (E-value = 0, at position 88,801,370 bp), justifying the association with SSR_2-1, which is located on the same chromosome as much as 15,695,145 bp upstream. Although neither the SCAR119 marker locus nor the 2.1 and 5.5 SSR marker loci are located on the sex chromosome (i.e., the chromosome in *Cannabis* that determines the sex of the flower), it is likely that specific regions of autosomal chromosomes also contribute to sex determination. In this regard, a recent study has reported that, of 555 sex-linked genes, 363 mapped to sex chromosomes, while the remaining 192 (i.e., 35% of all sex-linked genes) mapped to all the other autosomes [52].

## 3. Materials and Methods

### 3.1. Plant Materials of Cannabis

Fresh samples from 104 individuals representing 11 different *Cannabis sativa* industrial hemp varieties of high breeding value kindly provided by Gruppo Padana (Paese, TV, Italy) were collected in December 2020 and stored at −20 °C. Varieties were selected based on their geographical origin (Italy, Finland, Hungary, France, Poland, and Netherlands) and their sex behavior (dioecious and monoecious). Detailed information is presented in Table 4.

Genomic DNA (gDNA) was extracted from 70–100 mg of each sample using the “DNeasy^®^ 96 Plant Kit” (Qiagen, Hilden, Germany) following the manufacturer’s instructions. The quality of the genomic DNA samples was assessed by electrophoresis on a 1% (*w*/*v*) agarose gel stained with 1X SYBR^®^ Safe™ DNA Gel Stain (Life Technologies, Carlsbad, CA, USA) in Tris–acetate-EDTA (TAE) running buffer. The yield and purity were evaluated using a NanoDrop 2000c UV–vis Spectrophotometer (Thermo Scientific, Pittsburgh, PA, USA). Following DNA quantification, all the DNA samples were diluted to a final concentration of 20 ng/μL to be used as templates for PCR amplification.

### 3.2. Analysis of the SSR Marker Loci

The amplification reactions were performed using the three-primer strategy reported by Schuelke [53] with some modifications. Briefly, for each primer pair, universal sequences (namely, M13 and PAN1–3) were used to tag the 5′ end of the forward primer and were utilized in PCR assays in combination with M13, PAN1, PAN2, and PAN3 fluorophore-labeled oligonucleotides. The fluorophores used in all amplification reactions were 6-FAM, VIC, NED, and PET. As a preliminary step, 41 SSR primer pairs previously designed by [9] based on a de novo SSR identification analysis on the cs10 genome were evaluated to amplify a subset of DNA samples (at least one for each population). Each PCR contained Platinum Mix 2 × (Applied Biosystems, Carlsbad, CA, USA), GC Enhancer 10 ×, 0.25 µM tailed forward primer, 0.75 µM reverse primer, 0.5 µM fluorophore-labeled oligonucleotides, 40 ng of DNA template, and H2O to a final volume of 20 µL. Reactions were performed in a Veriti^®^ 96 Well Thermal Cycler (Applied Biosystems^®^, Carlsbad, CA, USA) as follows: 95 °C for 5 min; 35 cycles at 95 °C for 30 s, 55 °C (or 57 °C) for 45 s and 72 °C for 45 s; and a final extension of 30 min at 60 °C. The quality of the PCR products was assessed by electrophoresis on a 2% (w/v) agarose gel stained with 1X SYBR^®^ Safe™ DNA Gel Stain (Life Technologies, Carlsbad, CA, USA) using Tris–acetate-EDTA (TAE) running buffer. DNA fragment capillary electrophoresis was then performed with an ABI PRISM 3130xl Genetic Analyzer (Thermo Fisher, Pittsburgh, PA, USA) using LIZ500 (Applied Biosystems, Carlsbad, CA, USA) as the molecular weight standard. Fragment analysis was conducted using Peak-Scanner V1.0 (Applied Biosystems, Carlsbad, CA, USA). A subset of 20 primer pairs was selected based on their amplification capability, polymorphism and repeatability. These primer pairs were organized into four multiplexes (Table 5) and then used to amplify all 104 genomic DNA samples using the same conditions previously reported.

### 3.3. Molecular Data Analysis

Statistical analyses for all SSR marker loci were performed using the PopGene software package v. 1.32 [54]. The observed number of alleles per locus (n_e_), Levene’s observed heterozygosity (H_o_; [55]), Nei’s expected heterozygosity (H_e_; Nei, 1973) and the average heterozygosity (H_a_; Nei, 1978) were computed for each SSR locus and over all SSR markers. PopGene software was also used to estimate F-statistics [56], including the heterozygosity within (F_is_) and between (F_it_) subpopulations as well as the fixation index (F_st_), according to Wright [57]. The phenotypic diversity of the allele profiles was estimated using Shannon’s information index (I) as reported by Lewontin [58]. Gene flow (N_m_) estimates among subpopulations were derived from the fixation index as described by McDermott and McDonald [59].

The genetic similarity (GS) in all the pairwise comparisons and the principal component analysis (PCoA) for the two main dimensions were conducted under default settings using NTSYS software package v. 2.21c [60]. Moreover, the cluster dendrogram was built based on the unweighted pair-group with arithmetic mean (UPGMA) method by applying Dice’s coefficient [61], and a bootstrap analysis was conducted with 1,000 resampling replicates [62] using Past3 software [63].

The population structure of the *C. sativa* collection was investigated using the model-based (Bayesian) clustering algorithm implemented in STRUCTURE software v.2.2 [64], which groups individuals according to marker allele combination and distribution. The number of founding groups ranged from 1 to 15, and the method described by [45] was used to evaluate the most likely estimation of K. A burn-in of 2 × 10^5^ and a final run of 10^6^ Markov chain Monte Carlo (MCMC) steps were set. GENALEX v 6.503 [65] was used to investigate the total genetic variation by means of analysis of molecular variance (AMOVA) and to evaluate the presence of private alleles among populations. Alleles were considered private when present with at least 5% frequency in one group and absent in all others.

### 3.4. Prediction of Plant Sex through the SCAR119 Marker and Linkage Disequilibrium Analysis

To predict the sex of dioecious individuals (61 out of 104) at the seedling stage, [35] developed a SCAR-based assay that generates 119 bp bands only in male plants. To test the reliability of this molecular assay, we applied this analysis to the entire germplasm by using the protocol described by the authors and the following oligos: SCAR119_F: 5′-TCAAACAACAACAAACCG-3′ and SCAR119_R: 5′-GAGGCCGATAATTGACTG-3′.

Electrophoresis was performed using a 1.5% agarose gel on a Mupid^®^-one apparatus (Advance^®^) with Tris-acetate-EDTA buffer (TAE) and SYBR Safe for staining. The presence and size of PCR products were visualized using an UV transilluminator (Uvitec Cambridge^®^).

Any possible linkage disequilibrium existing between SCAR119 and the SSR loci previously analyzed was estimated and tested by Fisher’s exact test. A probability lower than 0.01 was selected to indicate a statistically significant amount of disequilibrium. All calculations and analyses were conducted using Genetic Data Analysis (GDA) version 1.0 software [66].

## 4. Conclusions

*Cannabis sativa* is a high-demand crop with a long human history of medicinal, agricultural, industrial, and recreational uses. However, there is a lack of knowledge of this valuable plant in many fields of science, including the use of genomics for breeding new varieties and tracing their commercial derivatives.

Our research focused on the implementation and validation of an informative, reliable, and reproducible multilocus genotyping system in *C. sativa* that may be useful both for marker-assisted breeding of new varieties and for unequivocally identifying specific varieties already on the market or assessing their genetic identity. The availability of easy-to-use molecular tools for the authentication of newly released varieties is also crucial for protecting plant breeders’ rights and *Cannabis* derivatives’ consumers.

First, we demonstrated the effectiveness of our novel set of SSR marker loci for assessing the genetic distinctiveness, uniformity, and stability of individual varieties as well as for estimating heterozygosity/homozygosity statistics of single plants or populations and the extent of genetic variation within and genetic relationship among different *C. sativa* varieties. This information could be exploited for planning crosses and predicting heterosis as an expression of hybridity in experimental F1 populations based on the allelic divergence and genetic distance of the selected parental lines.

Finally, our study on linkage disequilibrium demonstrated a correlation between two newly developed SSR marker loci and the well-known sex determination locus, SCAR119, which may assist further investigations on dioecy in *C. sativa*.

Knowing the parental genotypes would enable not only protection of newly registered varieties but also assessment of the genetic purity and identity of the seed stocks of commercial F1 hybrids and certification of the origin of clonal samples (cuttings) of unknown derivation. Moreover, the newly developed panel of SSR markers not only discriminated southern European samples (Italian and Hungarian Origin) from northern European samples (France, Poland, Netherlands, and Finland) but also assigned each sample to a single variety of hemp or its country of origin with only a few exceptions (<5%).

We are confident that the DNA-based labeling of varieties covered by patent protection represents the only solution in the *Cannabis* market for managing intellectual property rights in the *Cannabis* seed industry and protecting professional producers of hemp cuttings and derivatives.

## Figures and Tables

**Table 1:** Global genetic diversity statistics sorted by locus related to 104 *Cannabis* individuals based on 20 SSR marker loci.

Locus	General Statistics			H-Statistics			**F-Statistics**			**Nm**
	N_a_	p_i_	PIC	H_o_	H_e_	H_a_	F_is_	F_it_	F_st_	
SSR_6–3	7	0.54	0.57	0.50	0.62	0.25	0.00	0.22	0.22	0.86
SSR_2–2	7	0.55	0.58	0.42	0.63	0.21	0.16	0.37	0.25	0.60
SSR_X-1	28	0.13	0.91	0.53	0.92	0.25	0.42	0.46	0.07	1.40
SSR_4–2	11	0.43	0.70	0.22	0.74	0.10	0.73	0.76	0.10	0.69
SSR_2–3	24	0.25	0.88	0.70	0.89	0.34	0.18	0.24	0.08	1.69
SSR_7–3	17	0.21	0.88	0.83	0.89	0.41	0.03	0.10	0.07	1.67
SSR_3–3	23	0.20	0.90	0.70	0.91	0.34	0.19	0.26	0.09	1.47
SSR_2–1	7	0.77	0.35	0.07	0.38	0.03	0.74	0.84	0.37	0.23
SSR_4–1	11	0.70	0.47	0.20	0.50	0.09	0.68	0.72	0.13	0.42
SSR_8–2	20	0.17	0.90	0.84	0.91	0.39	0.07	0.16	0.10	1.33
SSR_5–2	21	0.14	0.91	0.32	0.92	0.13	0.67	0.73	0.17	0.37
SSR_6–1	14	0.18	0.86	0.35	0.88	0.14	0.64	0.69	0.14	0.78
SSR_X-3	25	0.13	0.92	0.70	0.93	0.29	0.31	0.37	0.10	0.87
SSR_1–4	8	0.62	0.56	0.57	0.59	0.26	0.16	0.21	0.06	2.78
SSR_3–1	15	0.16	0.88	0.52	0.90	0.22	0.45	0.53	0.14	1.05
SSR_8–4	17	0.16	0.88	0.41	0.90	0.18	0.56	0.61	0.12	0.78
SSR_9–4	17	0.23	0.88	0.63	0.89	0.27	0.35	0.41	0.09	1.24
SSR_1–1	18	0.23	0.87	0.36	0.88	0.14	0.63	0.69	0.15	0.63
SSR_5–5	8	0.70	0.46	0.08	0.49	0.03	0.86	0.89	0.21	0.51
SSR_6–4	3	0.67	0.40	0.38	0.47	0.17	0.29	0.42	0.17	1.05
Mean	15.05	0.36	0.74	0.47	0.76	0.21	0.41	0.48	0.14	1.02
St. Dev.	7.12	0.23	0.20	0.23	0.19	0.11	0.27	0.24	0.08	0.13

N: sample size of individual genotypes; pi: frequency of the most common marker allele; Na: number of observed alleles per locus; PIC: polymorphism information content; H_o_: observed heterozygosity; H_e_: expected heterozygosity; H_a_: average heterozygosity; F_is_ and F_it_: Wright’s inbreeding coefficients; F_st_: fixation index; N_m_: gene flow.

**Table 2:** Descriptive genetic diversity statistics of the *Cannabis* accessions calculated for individual varieties, including heterozygosity degrees, similarity degrees, inbreeding coefficients, and gene flow estimates.

Variety	N	General Statistics						H-Statistics			F-Statistics			N_m_
		P	I	SM (%)	GS (%)	N_o_	N_e_	H_o_	H_e_	H_a_	F_is_	F_it_	F_st_	
ITA1	10	0.90	1.19 ± 0.13	65.68 ± 4.42	88.68 ± 1.71	4.90 ± 0.56	3.11 ± 0.32	0.59 ± 0.05	0.62 ± 0.06	0.26 ± 0.16	0.13	0.12	0.56	0.19
ITA2	9	0.95	1.15 ± 0.12	64.21 ± 4.80	89.16 ± 1.62	4.40 ± 0.48	3.15 ± 0.36	0.58 ± 0.05	0.63 ± 0.06	0.20 ± 0.17	0.27	0.36	0.68	0.12
ITA3	10	1.00	1.49 ± 0.09	67.96 ± 3.76	86.62 ± 1.55	6.15 ± 0.47	4.00 ± 0.36	0.70 ± 0.03	0.74 ± 0.03	0.25 ± 0.14	0.26	0.29	0.64	0.14
HUN1	11	1.00	1.15 ± 0.11	67.50 ± 5.67	89.96 ± 1.67	4.60 ± 0.46	3.03 ± 0.32	0.59 ± 0.05	0.62 ± 0.05	0.19 ± 0.13	0.31	0.40	0.70	0.11
HUN2	10	1.00	1.35 ± 0.13	67.36 ± 3.31	87.09 ± 1.47	5.75 ± 0.63	3.80 ± 0.49	0.64 ± 0.04	0.68 ± 0.05	0.22 ± 0.13	0.24	0.35	0.68	0.12
FIN	11	0.85	1.41 ± 0.17	69.76 ± 2.97	87.54 ± 1.27	6.15 ± 0.74	4.43 ± 0.55	0.63 ± 0.07	0.66 ± 0.07	0.22 ± 0.17	0.34	0.32	0.66	0.13
NED	13	0.90	1.15 ± 0.14	65.67 ± 5.88	89.37 ± 1.77	4.60 ± 0.50	3.29 ± 0.40	0.57 ± 0.06	0.59 ± 0.06	0.18 ± 0.15	0.35	0.38	0.69	0.11
POL	8	1.00	1.04 ± 0.12	64.16 ± 4.91	89.91 ± 1.34	3.95 ± 0.41	2.77 ± 0.32	0.54 ± 0.05	0.58 ± 0.05	0.21 ± 0.16	0.22	0.26	0.63	0.15
FRA1	10	0.95	1.22 ± 0.14	63.57 ± 6.13	88.27 ± 2.15	4.70 ± 0.51	3.57 ± 0.42	0.60 ± 0.06	0.64 ± 0.06	0.18 ± 0.16	0.31	0.45	0.73	0.09
FRA2	9	1.00	1.39 ± 0.12	63.36 ± 4.30	86.26 ± 1.81	5.45 ± 0.56	4.02 ± 0.50	0.67 ± 0.04	0.72 ± 0.04	0.21 ± 0.13	0.32	0.40	0.70	0.11
FRA3	3	0.65	0.56 ± 0.11	49.86 ± 9.43	91.08 ± 2.13	2.10 ± 0.25	1.89 ± 0.21	0.34 ± 0.06	0.48 ± 0.09	0.17 ± 0.16	0.02	0.09	0.54	0.21

Variety: code identifying the *Cannabis* variety; N: number of individuals per variety; P: proportion of polymorphic loci (P = npj/ntot); I: estimates of Shannon’s information index of phenotypic diversity; Simple matching-based genetic similarity (SM): the genetic similarity coefficient or simple matching coefficient calculated within each variety without considering all the others; MGS: mean genetic similarity calculated using simple matching coefficient calculated considering all the varieties; N_o_: average number of observed alleles per locus; N_e_: effective number of alleles; H_o_: observed heterozygosity; H_e_: expected heterozygosity; H_a_: average heterozygosity; F_is_ and F_it_: Wright’s inbreeding coefficients; F_st_ = fixation index; N_m_ = gene flow.

**Table 3:** Pairwise linkage disequilibria (LD) between all SSR marker loci and the SCAR119 marker. Significant LD **: 0.001 < *p*<0.01 ***: *p* < 0.001.

	SCAR	SSR_ 1–1	SSR_ 1–4	SSR_ 2–1	SSR_ 2–2	SSR_ 2–3	SSR_ 3–1	SSR_ 3–3	SSR_ 4–1	SSR_ 4–2	SSR_ 5–2	SSR_ 5–5	SSR_ 6–1	SSR_ 6–3	SSR_ 6–4	SSR_ 7–3	SSR_ 8–2	SSR_ 8–4	SSR_ 9–4	SSR_ X-1	SSR_ X-3
**SCAR**	×			**								**									
**SSR_1–1**	0.025	×		**			**			**		**						**	**	**	
**SSR_1–4**	0.533	0.167	×																		
**SSR_2–1**	0.008	0.002	0.031	×	**	**	**		**	**	**	***	**		**	**	**		***	***	
**SSR_2–2**	0.115	0.062	0.097	0.003	×				**	**		**	**								
**SSR_2–3**	0.900	0.032	0.871	0.002	0.410	×															
**SSR_3–1**	0.092	0.005	0.053	0.003	0.029	0.132	×			***	**	***	***			***		**	**	***	**
**SSR_3–3**	0.103	0.036	0.254	0.051	0.121	0.617	0.044	×	**	**		**							**		
**SSR_4–1**	0.014	0.019	0.215	0.008	0.005	0.056	0.013	0.006	×	***		**								**	**
**SSR_4–2**	0.014	0.005	0.015	0.002	0.002	0.194	<0.001	0.002	<0.001	×	***	***	**	**		**		***	**		**
**SSR_5–2**	0.058	0.013	0.227	0.005	0.021	0.238	0.009	0.030	0.016	<0.001	×	**						**			**
**SSR_5–5**	0.003	0.002	0.016	<0.001	0.001	0.014	<0.001	0.005	0.003	<0.001	0.001	×	**	***	**	***	**	**	***	***	**
**SSR_6–1**	0.028	0.048	0.026	0.004	0.004	0.082	<0.001	0.046	0.042	0.004	0.011	0.002	×		**	**			**	**	
**SSR_6–3**	0.253	0.135	0.916	0.028	0.103	0.484	0.308	0.342	0.018	0.003	0.037	<0.001	0.021	×							
**SSR_6–4**	0.089	0.012	0.333	0.007	0.047	0.790	0.100	0.111	0.063	0.020	0.118	0.002	0.007	0.294	×						
**SSR_7–3**	0.285	0.016	0.518	0.006	0.019	0.138	<0.001	0.090	0.014	0.001	0.015	<0.001	0.002	0.243	0.100	×				**	
**SSR_8–2**	0.806	0.019	0.486	0.006	0.078	0.170	0.051	0.107	0.122	0.014	0.026	0.010	0.120	0.369	0.431	0.168	×				
**SSR_8–4**	0.060	0.003	0.058	0.011	0.078	0.042	0.001	0.019	0.015	<0.001	0.007	0.009	0.032	0.113	0.069	0.040	0.085	×	**	**	**
**SSR_9–4**	0.083	0.008	0.070	<0.001	0.126	0.250	0.002	0.010	0.019	0.002	0.046	<0.001	0.008	0.144	0.162	0.225	0.447	0.010	×	**	
**SSR_X-1**	0.035	0.006	0.180	<0.001	0.102	0.041	<0.001	0.084	0.003	0.016	0.057	<0.001	0.007	0.063	0.132	0.003	0.145	0.007	0.003	×	
**SSR_X-3**	0.205	0.034	0.529	0.018	0.160	0.380	0.007	0.024	0.003	0.003	0.004	0.001	0.015	0.197	0.350	0.051	0.209	0.004	0.184	0.015	×

**Table 4:** Detailed information of the 104 samples analyzed in this study. The name of each variety, the geographical origin, the number of samples and the sex behavior are reported along with a representative picture of the leaf morphology.

Variety	Origin	N. of Samples	Sex Behavior of the Samples			Leaf
			Dioecious (Male)	Dioecious (Female)	Monoecious	
ITA1	Italy	10	7	3		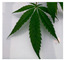
ITA2	Italy	9	4	5		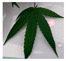
ITA3	Italy	10	2	8		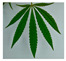
HUN1	Hungary	11	6	5		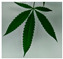
HUN2	Hungary	10	5	5		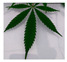
FIN	Finland	11	3	8		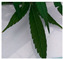
NED	Netherlands	13			13	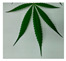
POL	Poland	8			8	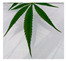
FRA1	France	10			10	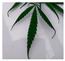
FRA2	France	9			9	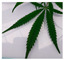
FRA3	France	3			3	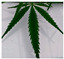

**Table 5:** Information on the 20 microsatellite markers validated using a core collection of high breeding value plant materials of *Cannabis*.

Locus Name	Start	End	Expected Size	Multiplex	Fluo Dye	Ta (°C)	Motif	Forward Primer	Reverse Primer
SSR_6–3	35,062,092	35,062,261	180–200	1	M13	55	(AAT)10	ATCTCATTTTCCGTACCTGTT	CTAATTCTCAACTTAACCGCG
SSR_2–2	27,019,093	27,019,345	250–270	1	M13	55	(TGA)12	TAGTAGTAGTAGTGCCTGAGG	ACCTTAACAACACCACAACTA
SSR_X-1	12,090,959	12,091,352	390–450	1	M13	55	(TC)40	TTGTCAAGGGAGCTTAGTTAG	ATGTGTATTTCTCGCCTGTTA
SSR_4–2	38,738,240	38,738,472	230–260	1	PAN1	55	(AT)17	CAGAGTTTGGTCCTTTTCAAA	CACGGATTTTAAGCATTGGAT
SSR_2–3	49,240,375	49,240,744	350–410	1	PAN1	55	(GA)22	CTCCCTGCCATTAGACAAATA	CCAGGAGGTAATTTTCTGCTA
SSR_7–3	51,776,452	51,776,692	230–280	1	PAN2	55	(CT)22	ACTGTGAACTGTCCTTTTACA	AACAACCTGAAATCCGAAAAG
SSR_3–3	59,258,629	59,258,880	250–300	1	PAN3	55	(AG)21	CAAAGAAAGCAGGCATTAGTT	CTCTCTGTGAATGTGATCTGT
SSR_2–1	15,695,145	15,695,388	240–260	2	M13	55	(AAT)11	GGCAGGAAAAATCTCAAACAT	ACATTGGAATTAGACAGAGCA
SSR_4–1	3,414,697	3,414,947	230–270	2	PAN1	55	(ATA)21	GTTGGTTATGTGTTAGGGTCT	GTTATGGACAAACAATGCATG
SSR_8–2	13,924,026	13,924,199	180–220	2	PAN2	55	(CT)21	CATCACACCAGGTACCAATAT	CATGAAACAACGTTGGGTTAT
SSR_5–2	34,558,385	34,558,643	250–300	2	PAN2	55	(CT)32	TGGCTGAAAGTAAGAAAAGAC	TTATCGCTCAAAACACTCAAC
SSR_6–1	3,764,859	3,765,058	200–270	2	PAN3	55	(AT)17	ACTTCACATGAGATTGAGAACA	TCCTTTGGATTCATTAAGTTGT
SSR_X-3	71,305,129	71,305,410	280–350	2	PAN3	55	(TC)41	ACAGTAGTTTTCAGGGTTGAA	TCACACCAATATCTATCAGCC
SSR_1–4	86,039,144	86,039,328	180–220	3	M13	55	(TTA)17	TCAAGTTACGTAATCCCCAAA	CCTAAGCACAAGGTTAAATCAT
SSR_3–1	12,247,530	12,247,829	300–340	3	M13	55	(TC)32	TGATTTTGCGACCCTTTTATG	CTTTTGCAGGTACATCCAAAA
SSR_8–4	50,925,135	50,925,396	280–330	3	PAN1	55	(TC)22	TATGCATCCATTGTACCTGTT	TAATGTTTGTGTGTGTGCAAA
SSR_9–4	58,895,568	58,895,670	110–150	4	PAN1	57	(CT)16	TTTCCTGCTCACCTTAAACC	AACCTATATTGAGACGAACCG
SSR_1–1	12,756,851	12,757,030	180–220	4	PAN1	57	(TC)33	AAACTGACAGCTTAAGCATTC	TGGGCATGTACTCTATCACTA
SSR_5–5	82,565,436	82,565,719	270–290	4	PAN2	57	(GA)18	AGAGGAAGGAAAGAGAGCTAT	CACGAGGGAGCCTTATTAATA
SSR_6–4	63,517,285	63,517,456	170–180	4	PAN3	57	(CT)30	ACGAGACTTTACAGAGAACAA	AGATAGGGAAGAACACAACAC

## Data Availability

The data presented in this study are available within the article or as Supplementary Material.

## Supplementary Materials

The following Supplementary Materials are available online at https://www.mdpi.com/article/10.3390/plants10102174/s1: Figure S1: Delta-K obtained following genetic structure analysis; Table S1: Detailed SSR profile information of the 104 samples; Table S2: Genetic similarity matrix; Figure S2: Cluster dendrogram based on unweighted pair-group with arithmetic mean (UPGMA) method by applying Dice’s coefficient.
