# Supplementary figures and images for: Developing and Testing Molecular Markers in Cannabis sativa (Hemp) for Their Use in Variety and Dioecy Assessments
Source: Plants (Basel). 2021 Oct 14;10(10):2174. doi: 10.3390/plants10102174 (PMC8540786; doi:10.3390/plants10102174)

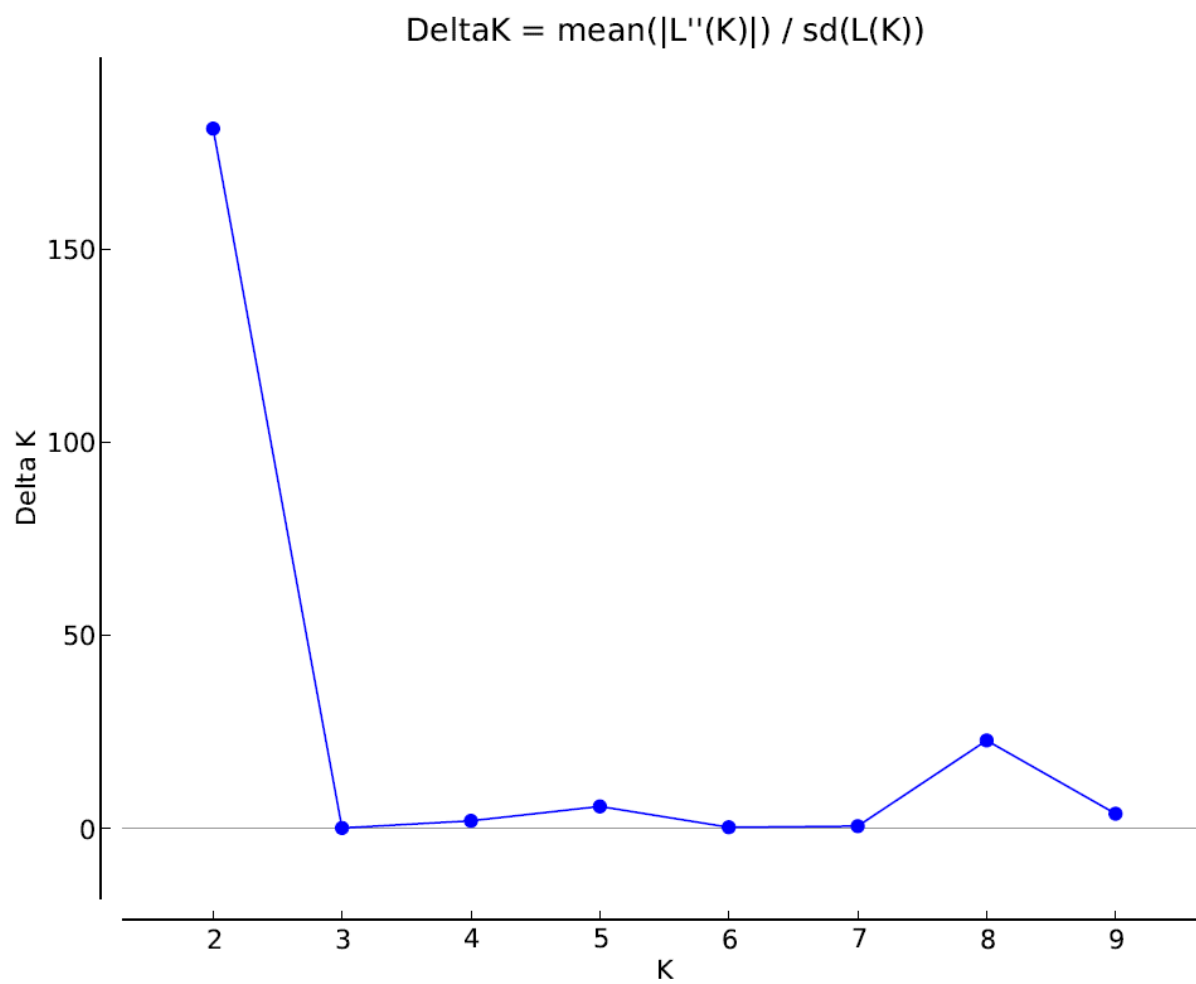

**Supplementary figure S1:** Delta K obtained following the genetic structure analysis.

Supplement: Supplementary file 1 [file plants-10-02174-s001.zip › Supplementary figure S1 DeltaK.pdf]

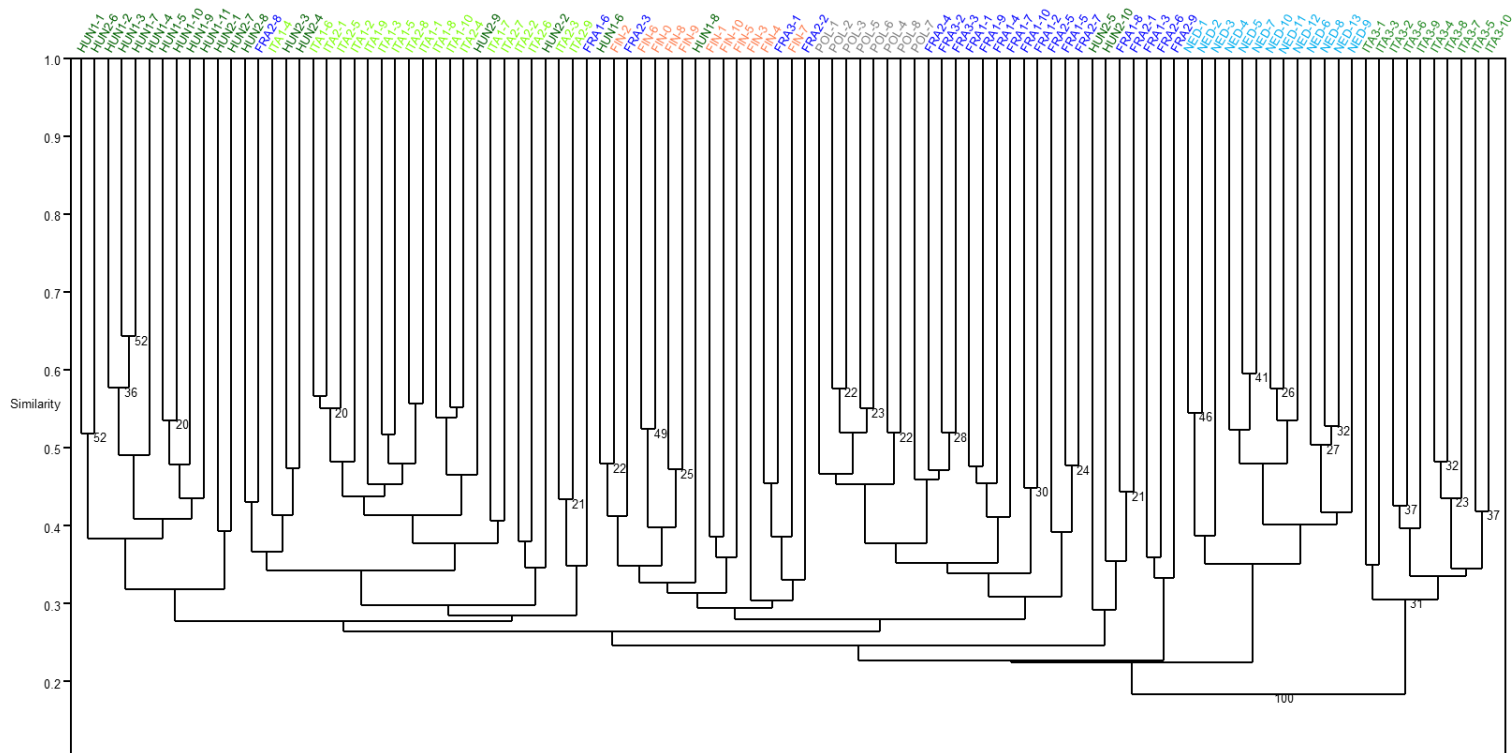

Supplement: Supplementary file 1 [file plants-10-02174-s001.zip › Supplementary figure S2 UPGMA.pdf]
